# Effects of vitamins A and E on eprinomectin transport in Caco-2 cells

**DOI:** 10.17221/8/2026-VETMED

**Published:** 2026-08-21

**Authors:** Yigit Gunes, Yakup Gultekin, Osman Dogan, Ceren Anlas, Oya Ustuner, Nurcin Yilmaz, Tulay Bakirel

**Affiliations:** ^1^Department of Pharmacology and Toxicology, Faculty of Veterinary Medicine, Istanbul University-Cerrahpasa, Istanbul, Turkiye; ^2^Department of Pharmaceutical Technology, Faculty of Pharmacy, Selcuk University, Konya, Turkiye; ^3^Department of Pharmaceutical Technology, Faculty of Pharmacy, Hacettepe University, Ankara, Turkiye; ^4^Department of Pharmaceutical Technology, Faculty of Pharmacy, Baskent University, Ankara, Turkiye

**Keywords:** Caco-2 transwell model, eprinomectin, fat-soluble vitamins, intestinal permeability, P-glycoprotein

## Abstract

Eprinomectin (EPR) is a macrocyclic lactone antiparasitic widely used in veterinary medicine. Although the off-label oral use of the pour-on formulation of EPR is known to be effective and practical, data regarding its interactions with foods or vitamin supplements that may affect intestinal absorption remain limited. This study investigated the effects of vitamin A (VA) and vitamin E (VE) on the transepithelial permeability of EPR using the Caco-2 cell monolayer model. An analytical method for EPR quantification was developed using ultra-performance liquid chromatography tandem mass spectrometry (UPLC-MS/MS). The integrity of tight junction dynamics in epithelial monolayers was monitored using transepithelial electrical resistance (TEER) throughout the 21-day differentiation protocol. Apparent permeability coefficients (*P*_app_) were calculated in both apical-to-basolateral and basolateral-to-apical directions and compared between EPR alone and EPR combined with the vitamins. EPR alone exhibited moderate permeability across Caco-2 monolayers (7.64 ± 2.27 × 10^–6^ cm/s). Notably, our results demonstrate that co-treatment of EPR with VA alone, as well as co-treatment with the combination of VA and VE, significantly reduced EPR permeability *in vitro* (*P* < 0.001). These findings highlight possible food/nutrient-drug interactions relevant to veterinary oral drug administration, and additional mechanistic and *in vivo* studies will be necessary to clarify the underlying pathways.

Anthelmintic macrocyclic lactones (MLs) are antiparasitic drugs with broad-spectrum activity, favourable safety profiles, and ease of administration (Campbell 2012; Geary and Moreno 2012; Mohammed and Ahmed 2024), and are widely used in both animals (McKellar and Gokbulut 2012) and humans (Molyneux et al. 2003). Eprinomectin [4''-epiacetylamino-4''deoxyavermectin B1], a member of the avermectin class of MLs, is used for the treatment of internal and external parasites in animals such as cattle, sheep, goats, camels, pigs, rabbits, and dogs (Figure 1) (EMEA 1996; Hinney et al. 2022). Binding of eprinomectin (EPR) to glutamate-gated chloride channels increases chloride conductance across the cell membrane, resulting in hyperpolarisation and flaccid paralysis of parasites (Stern et al. 2025).

**Figure 1:**
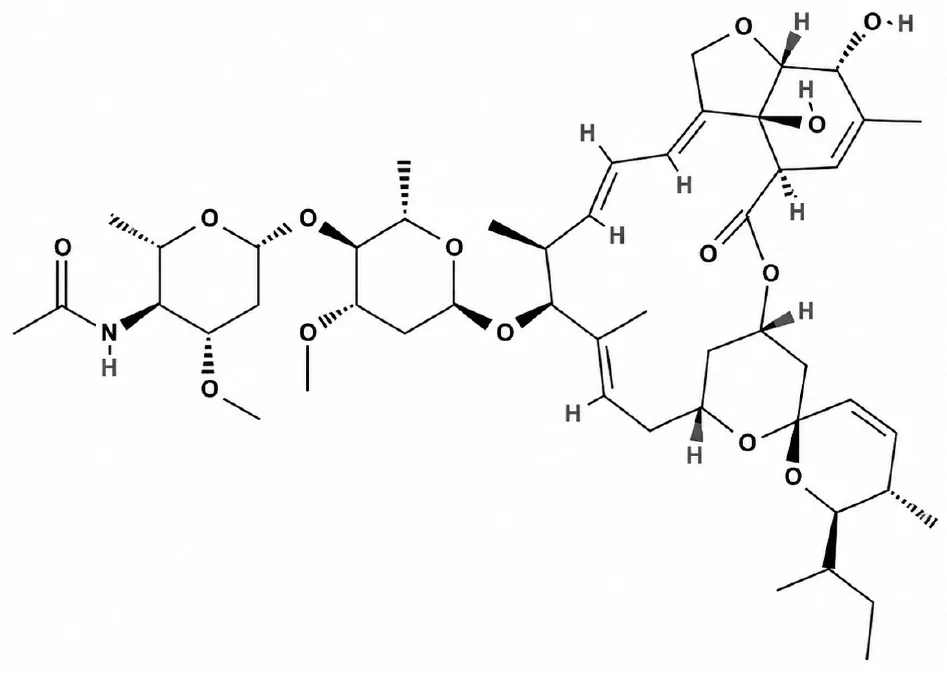
Molecular structure of eprinomectin

EPR has been approved as both a pour-on and a subcutaneous (S/C) injectable formulation in several countries and provides high therapeutic efficacy against a broad range of gastrointestinal nematodes and *Dictyocaulus filaria* lungworms in target animal species (Rehbein et al. 2014; Hamel et al. 2018). Several studies have shown that the pour-on administration of EPR has some limitations under field conditions (Murri et al. 2014; Rambozzi et al. 2025). In this context, several challenges have been documented, including the reduced bioavailability of EPR observed in lactating goats compared with dry goats, the need for higher dose rates in goats than in cattle (specifically 1.0 mg/kg b.w. instead of 0.5 mg/kg b.w.), and field observations suggesting inconsistent efficacy of topical EPR as assessed by post-treatment faecal egg count (FEC) reductions (Lespine et al. 2012; Murri et al. 2014; Bordes et al. 2022). On the other hand, it has been reported that oral administration of the pour-on formulation of EPR is a practical method of administration in goats (Rostang et al. 2020).

Although numerous studies have primarily emphasised the pour-on and S/C pharmacokinetic profiles of EPR in various animal species (Gokbulut et al. 2011; Rehbein et al. 2014; Miller et al. 2025), particularly for off-label use, pharmacokinetic data remain limited. Badie et al. (2015) determined that oral administration of the topical EPR formulation (0.5 or 1.0 mg/kg b.w.) to goats led to faster absorption and significantly higher peak concentrations (C_max_) in plasma and milk compared to the traditional pour-on route. This increased systemic availability was linked to high anthelmintic efficacy, demonstrating 100% effectiveness against *H. contortus* and 99.8% against *T. colubriformis* at the 0.5 mg/kg dose. To evaluate the potential of an orally administered drug as a rational therapeutic option, it is essential to fully characterise the influence of the nutritional status (fasted versus fed conditions) on its systemic exposure and drug delivery mechanisms (CDER 2002; DeHaven and Conner 2014). Fat-soluble vitamins, such as vitamins A and E, are routinely and widely used as nutritional additives in animal feeding (EFSA 2010; EFSA 2013). Certain fat-soluble vitamins have been reported to modulate the absorption of P-gp substrate drugs through the induction or inhibition of transmembrane transporter proteins, particularly P-gp and BCRP (Thomas 1995; Podszun and Frank 2014). These findings suggest that intestinal absorption is a key determinant of EPR systemic exposure when orally administered.

Recent studies have shown that some fat-soluble vitamins (A and E) can modulate the intestinal absorption of some drugs by interacting with P-gp, which may limit the absorption of orally administered drugs (Lespine et al. 2009; Husain et al. 2022). For instance, d-α-tocopheryl polyethylene glycol succinate (TPGS), an FDA-approved pharmaceutical adjuvant, is recognised as a potent P-gp inhibitor that enhances the intracellular accumulation of substrates (Yang et al. 2018; Husain et al. 2022). Similarly, vitamin A palmitate (VA palmitate), a GRAS compound, has been confirmed to inhibit the P-gp transporter; it significantly increased the permeability of P-gp substrates (Reker et al. 2020). In addition to transporter-mediated effects, fat-soluble vitamins may also influence drug permeability through physicochemical interactions at the intestinal epithelial membrane. EPR, as a lipophilic compound, is particularly susceptible to such interactions (Lespine et al. 2009). Due to their lipophilic nature, vitamins A and E can partition into the lipid bilayer, where they may alter membrane fluidity and lipid organisation and potentially sequester lipophilic drug molecules within the membrane, thereby influencing their availability and diffusion at the apical interface (Atkinson et al. 2021; Radzin et al. 2023). These membrane-level interactions may affect transcellular diffusion independently of classical efflux transporter modulation (Wang and Quinn 2000). Furthermore, fat-soluble vitamins may influence P-gp activity through both direct and indirect mechanisms. Direct effects may include inhibition of P-gp ATPase activity or competition at substrate-binding sites, whereas indirect effects may arise from alterations in membrane fluidity and lipid microenvironment that can modify P-gp conformation and function. Additionally, fat-soluble vitamins may regulate P-gp expression via nuclear receptor-mediated pathways, such as the pregnane X receptor (PXR), thereby affecting the transport of P-gp substrates such as EPR (Lespine et al. 2009; Husain et al. 2022).

Supporting the relevance of nutrient–drug interactions involving macrocyclic lactones, an *in vivo* study confirmed that high oral doses of vitamins A and E, as well as their combination, significantly increased the plasma and brain concentrations of ivermectin, a well-established P-gp substrate, thereby suggesting a transporter-mediated modulation of drug disposition (Tras et al. 2023). Such vitamin-mediated modulation of P-gp highlights the relevance of investigating whether similar mechanisms affect the intestinal transport of EPR. Beyond formulation- and species-related factors, therefore, intestinal transport mechanisms play a critical role in determining the systemic availability of orally administered macrocyclic lactones. In this context, EPR is a known substrate of ATP-binding cassette transporters, particularly P-gp, which limits transepithelial drug absorption at the intestinal epithelium (Kiki-Mvouaka et al. 2010).

Despite its widespread use, the interactions of EPR with vitamins during transepithelial transport have not been fully elucidated. Our study aimed to investigate the effects of VA and VE, two of the most frequently supplemented micronutrients in livestock and horses (McDowell 2006; Verhoeckx et al. 2015), on the transepithelial transport of EPR. To this end, the Caco-2 cell monolayer model, which is widely recognised for its close correlation with *in vivo* intestinal absorption and for its expression of major efflux and uptake transporters, was employed to assess EPR permeability. Subsequently, EPR concentrations were quantified using a validated UPLC-MS/MS method to ensure sensitive and accurate analytical evaluation. To the best of our knowledge, this is the first study to systematically evaluate how fat-soluble vitamins affect the intestinal permeability of EPR.

## MATERIAL AND METHODS

### Reagents and drugs

EPR reference standard (a mixture of component B1a ~95% and component B1b ~2.6%) (USP, Rockville, MD, USA), vitamin A propionate, and vitamin E were kindly donated by Ceva Animal Health (İstanbul, Türkiye). Dulbecco’s modified Eagle’s medium (DMEM) was obtained from Sigma Aldrich (St. Louis, MO, USA). Caco-2 cell line was supplied by the American Type Culture Collection (ATCC) (Manassas, VA, USA). Foetal bovine serum, penicillin/streptomycin solution (100×), trypsin (0.25%)-EDTA (0.02%) solution, and Hank’s balanced salt solution (HBSS) were obtained from Biochrom AG (Berlin, Germany). Lucifer yellow CH was purchased from Cayman Chemical Company (Ann Arbor, MI, USA). All other compounds used were commercially available reagent-grade chemicals.

### Cell culture

The permeability of EPR in combination with VA and VE was evaluated using the human colorectal adenocarcinoma cell line (Caco-2). Caco-2 cells between passages 35 and 42 were cultured at 37 °C in a humidified atmosphere containing 5% CO_2_. The cell culture medium contained DMEM supplemented with 10% (v/v) foetal bovine serum (FBS) and 1% penicillin/streptomycin solution (100 IU/ml penicillin and 100 μg/ml streptomycin). Upon reaching 80–85% confluence within 4–7 days, cells were detached using trypsin–EDTA and subsequently seeded onto polycarbonate membrane inserts (1 μm pore size, 12-well cell culture plate; Greiner Bio-One, Frickenhausen, Germany) at a density of 50 000 cells per well. The cells were cultured for 21–22 days to allow for monolayer formation. The culture medium was refreshed every other day, and monolayer integrity was monitored by measuring transepithelial electrical resistance (TEER) using a Millicell-ERS volt-ohm meter (Merck Millipore, Darmstadt, Germany).

### Permeability assay

**Figure 2:**
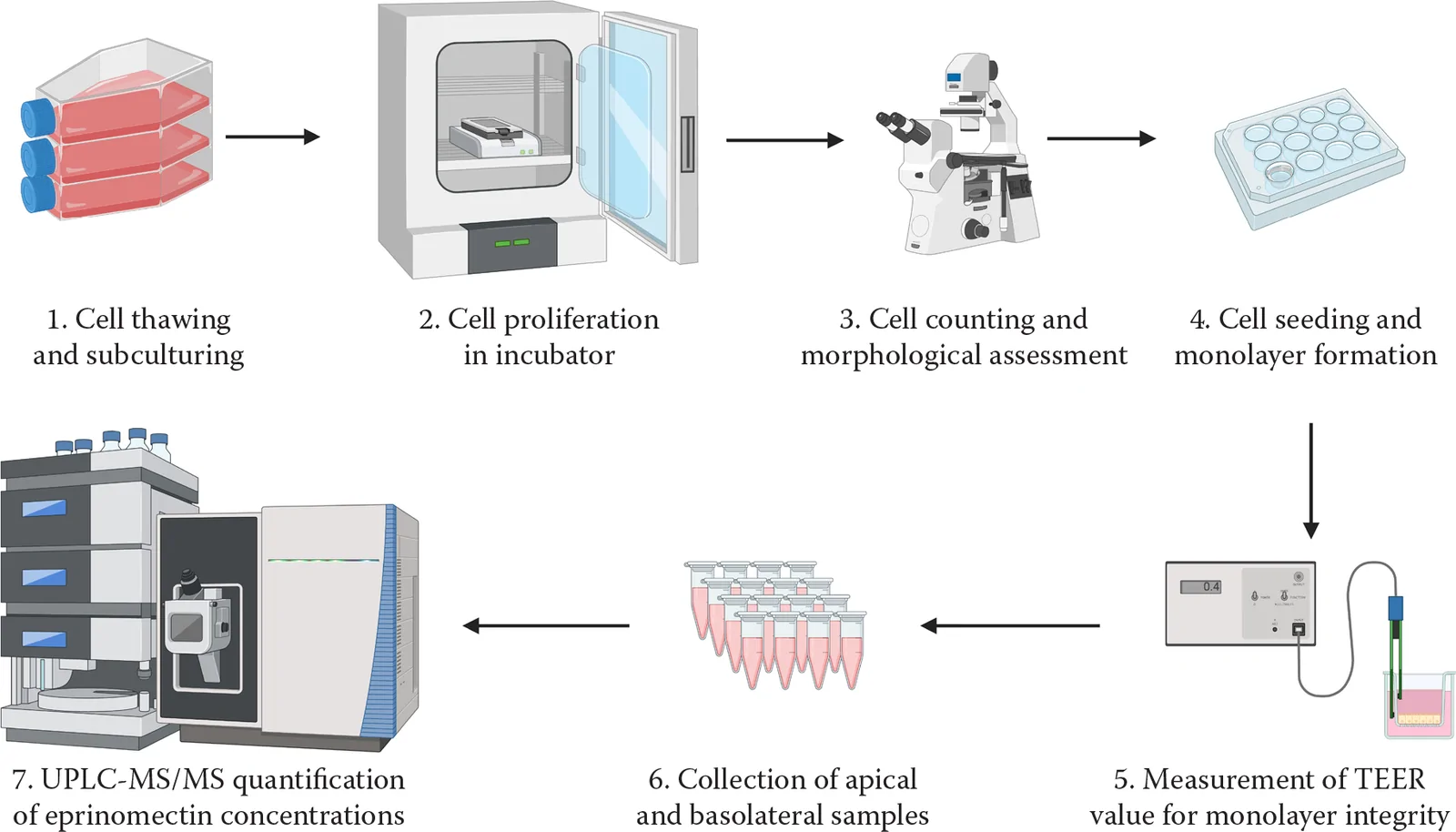
Schematic representation of the Caco-2 cell permeability assay for eprinomectin 1. Cell thawing and subculturing: Frozen Caco-2 cells were thawed and expanded under sterile conditions. 2. Cell proliferation in an incubator: Cells were maintained under standard culture conditions (37 °C, 5 % CO_2_, humidified atmosphere). 3. Cell counting and morphological assessment: Cell density and morphology were evaluated microscopically before seeding. 4. Cell seeding and monolayer formation: Cells were seeded onto permeable inserts and cultured until a confluent monolayer was formed. 5. Measurement of TEER values for monolayer integrity: Transepithelial electrical resistance (TEER) was measured to verify tight-junction integrity prior to the permeability assay. 6. Collection of apical and basolateral samples: After incubation with eprinomectin and/or vitamin treatments, samples were collected from both compartments. 7. UPLC-MS/MS quantification of eprinomectin concentrations: Eprinomectin concentrations in the collected samples were determined by liquid chromatography coupled with tandem mass spectrometry. Created in https://BioRender.com


Permeability studies were conducted using Caco-2 cells to evaluate the permeability of EPR alone and in combination with VA and VE. Metoprolol tartrate and lucifer yellow were used as positive and negative controls, respectively, to confirm monolayer integrity in accordance with ICH guidelines (ICH 2019). Cells were seeded onto 12-well Transwell^®^ inserts (pore size: 1 μm; surface area: 113.1 mm^2^). A total of 500 μl of cell suspension was added to the apical compartment, while 1 ml of fresh culture medium was added to the basolateral compartment. For the initial three days, the plates were maintained in an incubator (37 °C, 5% CO_2_) without any treatment to allow the cells to grow. On the following days, the culture medium was replaced with fresh medium every other day. On day 21, TEER values were measured to assess monolayer integrity. Only monolayers exhibiting TEER values between 400 and 600 Ω·cm^2^ were selected for the assay (Artursson and Karlsson 1991). Drug-free HBSS was added to both compartments and the cells were then incubated for 30 minutes. All permeability experiments were performed under serum-free conditions using HBSS. EPR (20 μg/ml) and its combinations with VA (33 μM) and VE (33 μM) were prepared in HBSS (Collnot et al. 2006). For the permeability test, 0.5 ml of the test solution was added to the apical compartment, and 1 ml of HBSS (drug-free) was added to the basolateral compartment. The inserts were incubated for 2 h at 37 °C (Figure 2). Following incubation, samples were collected and analysed by UPLC-MS/MS to quantify the amount of drug transported to the basolateral side. Apparent permeability coefficients (*P*_app_, cm/s) for EPR, EPR + VA, EPR + VE, and EPR + VA + VE were calculated using Equation 1.

$$P_{\text{app}}=[\text{d}Q/\text{d}t]\times [1/(A\times C_{0})]$$ (1)

where:

d*Q*/d*t* – cumulative transport rate (μM/min);

*A*  – surface area of inserts (cell monolayer) (cm^2^);

*C*_0_ – initial concentration in the apical region (μM).

### Sample preparation and UPLC-MS/MS analysis

To determine the transport rate of the test compound, an extraction procedure was performed prior to sample injection into the UPLC-MS/MS system. Calibration standards and quality control samples were prepared by spiking blank buffer. Subsequently, 50 μl of blank buffer was dispensed into two vials labelled as “blank” and “blank + IS” (internal standard). Then, 50 μl of the corresponding sample was transferred to the vials, followed by vortex mixing.

A volume of 0.15 ml of selamectin was added to all samples as the IS, and the mixtures were placed on a shaker for 5 minutes. The samples were centrifuged at 1 800 × *g* for 5 min at 20 °C. The supernatants were transferred into autosampler vials, and 5 μl of each was injected into the UPLC-MS/MS system (Figure 2).

The qualitative and quantitative determination of EPR was carried out using a Dionex Ultimate 3000 UPLC system coupled with a TSQ Fortis (Thermo Fisher Scientific Inc., Waltham, MA, USA) equipped with an ESI source operating in positive ionisation mode. The chromatographic separation was carried out using a Raptor Inert C18 column (50 × 2.1 mm, 2.7 μm; Restek, Bellefonte, PA, USA) with solvent A (5 mM ammonium formate + 0.1% formic acid) and solvent B (methanol + 0.1% formic acid). The following gradient was applied: 0–1.0 min, 5% B; 1.0–3.0 min, 5–90% B; 3.0–6.0 min, 90% B; and 6.0–8.0 min, 5% B for column re-equilibration. The flow rate, injection volume, and column temperature were 0.4 ml/min, 5 μl, and 40 °C, respectively. HESI interface conditions with multiple reaction monitoring (MRM) modes were spray voltage, static; sheath gas pressure, 45 arb; aux gas pressure, 15 arb; capillary temperature, 300 °C; and vaporiser temperature, 350 °C. Data were processed using TraceFinder v3.2 software (Thermo Fisher Scientific, Waltham, MA, USA).

### Calibration and method validation

Method validation was carried out by constructing 18** **independent calibration curves, each demonstrating linearity across the tested concentration range. The overall variance was assessed by calculating the relative deviation of each standard point from the regression curve. Standard deviations were less than 10% throughout the calibration range for EPR. At the LOQ (limit of quantification), accuracy (70–120%) and precision (CVs ≤20 %) were acceptable. In addition to LOQ evaluation, six replicate measurements were performed for six concentration levels (0.1, 0.25, 0.5, 1, 5, and 10 μg/ml). An accuracy range of 85–96% was obtained, and CVs of 25% under repeatability conditions were used to assess precision. Under reproducibility conditions, the LOQ accuracy of three fortified samples varied from 70% to 120%, with CVs of 15%. The limit of detection (LOD) and LOQ values for EPR were determined by the UPLC-MS/MS system and are presented in Table 1.

**Table 1:** Validation parameters of the analytical method for eprinomectin quantification

Parameters	EPR chromatographic conditions
Linearity range (μg/ml)	10–0.1
*R*^2^ value	0.993 6
LOD (ng/ml)	0.32
LOQ (ng/ml)	0.98
Intraday repeatability (%CV); *n* = 6	3.8
Interday repeatability (%CV); *n* = 6	7.4
Recovery (5 μg/ml, 0.5 μg/ml, 0.1 μg/ml)	96%, 94%, 85%
Retention time (min)	5.86

High recovery was obtained with the UPLC-MS/MS method; the *R*^2^ value was close to 1; the limits of detection and limits of quantification were low, and intraday to interday repeatability was <10%

CV = coefficient of variation; LOD = limit of detection; LOQ = limit of quantification

### Statistical analysis

Results are expressed as the mean ± SD. The significance of differences in the *P*_app_ values was determined by one-way analysis of variance (ANOVA), followed by Tukey’s multiple comparison test.

## RESULTS

**Figure 3:**
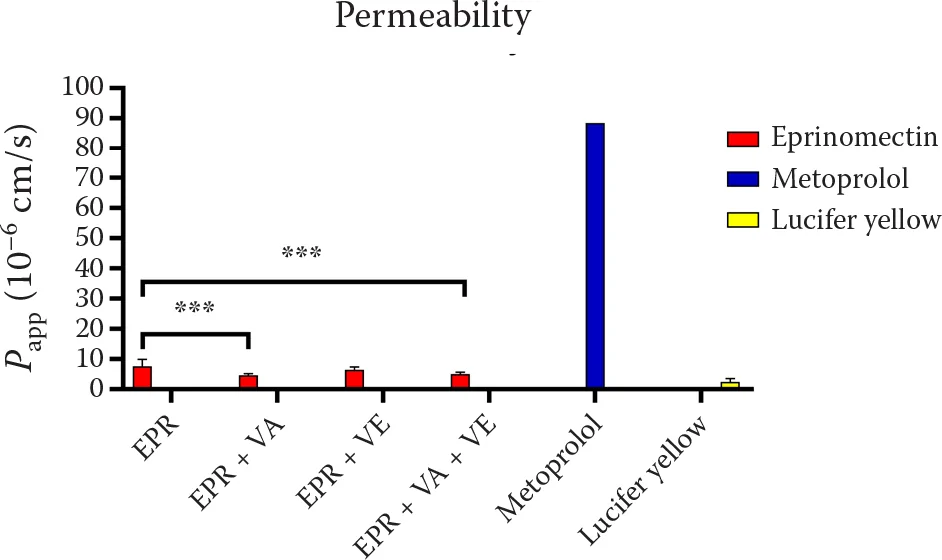
Apparent permeability coefficients (*P*_app_, ×10^–6^ cm/s) of eprinomectin (EPR) and its combinations with fat-soluble vitamins across Caco-2 cell monolayers Statistical significance was evaluated using one-way ANOVA followed by Tukey’s multiple comparison test (***P < 0.001). Data are expressed as mean ± SD (*n* = 3) Treatments included eprinomectin alone (EPR), eprinomectin with vitamin A (EPR + VA), eprinomectin with vitamin E (EPR + VE), and eprinomectin with both vitamins A and E (EPR + VA + VE). Metoprolol and Lucifer yellow were used as high- and low-permeability reference compounds, respectively

Permeability studies were conducted using Caco-2 cell monolayers to assess the transcellular transport of EPR (Figure 2). According to the results presented in Table 2 and Figure 3, the apparent permeability coefficients (*P*_app_) of EPR alone, EPR + VA, EPR + VE, EPR + VA + VE, and the control compounds were calculated.

**Table 2:** Transport parameters of eprinomectin across Caco-2 transwell model

Drug/formulation	*P*_app_ (10^–6^ cm/s)		
	EPR	Metoprolol	Lucifer yellow
EPR	7.64 ± 2.27	–	–
EPR + VA	4.58 ± 0.52***	–	–
EPR + VE	6.41 ± 0.93	–	–
EPR + VA + VE	5.06 ± 0.56***	–	–
Metoprolol	–	88.49 ± 1.42	–
Lucifer yellow	–	–	2.49 ± 1.03

Results were expressed as mean ± SD of three independent experiments in triplicate (*n* = 3). A significant decrease in *P*_app_ was observed with the addition of vitamin A alone and in combination with vitamin E (***P < 0.001 vs EPR alone). Metoprolol and Lucifer yellow were used as positive and negative controls for transport, respectively

EPR = eprinomectin; VA = vitamin A; VE = vitamin E

Metoprolol exhibited high permeability (88.49 ± 1.42 × 10^–6^ cm/s), whereas Lucifer yellow demonstrated low permeability (2.49 ± 1.03 × 10^–6^ cm/s) (Figure 3). EPR alone exhibited a mean *P*_app_ value of 7.64 ± 2.27 × 10^–6 cm/s^, indicating moderate permeability across Caco-2 monolayers. Co-treatment with VA significantly reduced EPR permeability to 4.58 ± 0.52 × 10^–6 cm/s^ (*P* < 0.001). The combination of VA and VE also markedly reduced EPR permeability (5.06 ± 0.56 × 10^–6^ cm/s, *P* < 0.001). Although a reduction in EPR permeability was seen with VE alone (6.41 ± 0.93 × 10^–6^ cm/s), the difference was not statistically significant. The analytical method applied, validated in accordance with current international guidelines (ICH 1996) (Table 1), enabled the selective and accurate identification and quantification of EPR. The chromatographic separation exhibited no overlap or interference at the retention time of EPR from matrix components or degradation products. Furthermore, no degradation products of EPR were detected using UPLC-MS/MS analysis at the end of the experiments.

## DISCUSSION

The reliability of the Caco-2 permeability assay was assessed through validation steps that confirmed the integrity of the monolayer and the suitability of the model. Monolayer integrity was controlled by ensuring TEER values of >300 Ω·cm^2^. Metoprolol, a high-permeability marker, exhibited significantly higher *P*_app_ values than EPR, while Lucifer yellow demonstrated limited paracellular transport, affirming monolayer integrity (Hidalgo et al. 1989; ICH 2019). These findings are consistent with previously reported data in the literature (Lennernas et al. 1996; Koljonen et al. 2006). Consequently, this indicates that the Caco-2 monolayers formed an intact and uniform monolayer epithelial barrier that effectively mimics the intestinal epithelium, thereby validating their reliability for use in *in vitro* permeability studies. LC-MS/MS analysis was performed on samples collected from both the apical and basolateral sides in the transport experiments to determine the permeability of EPR across the Caco-2 cell monolayer. The inherent high specificity and sensitivity of UPLC-MS/MS provided greater analytical accuracy than conventional HPLC-UV methods, primarily due to reduced chemical interference (Gunes et al. 2024).

Cellular permeability models in drug discovery and development allow the investigation of the permeability potential of drugs, the mechanism(s) of drug absorption, structure-transport relationships, and drug–food interactions (Han et al. 2006; Berginc et al. 2010; Volpe 2011; Awortwe et al. 2014). In the present study, the Caco-2 permeability model system was used to evaluate the modulatory effects of fat-soluble vitamins (VA and VE) on the intestinal permeability of EPR. Apparent permeability (*P*_app_) values serve as quantitative indicators of transcellular transport efficiency, with higher values associated with greater absorption potential (Volpe et al. 2007). Conversely, decreased *P*_app_ values may reflect restricted transport due to physicochemical factors or transporter modulation, including P-gp (Hidalgo et al. 1989; International Transporter Consortium et al. 2010). Our study determined that the co-administration of VA and EPR resulted in an approximately 40% decrease in the *P*_app_ value. VE did not significantly alter EPR permeability. Furthermore, when both VA and VE were combined, EPR permeability showed a ~34% reduction. Collectively, these findings suggest that the primary modulatory effect causing the reduction in EPR permeability is predominantly attributable to VA. This effect may be attributed to acute, non-genomic interactions occurring at the intestinal epithelial level (Losel et al. 2003). Due to its lipophilic ester structure, VA can rapidly partition into the membrane lipid phase, thereby altering membrane organisation or sequestering the freely diffusible fraction of EPR at the apical interface. Such membrane-associated interactions can limit transcellular diffusion without necessitating the direct modulation of efflux transporter activity (Artursson and Karlsson 1991; Herr et al. 1999; Volpe et al. 2007; Escriba et al. 2008). Taken together, these findings suggest that the observed decrease in EPR permeability is more likely driven by membrane-level physicochemical interactions rather than direct modulation of P-gp–mediated efflux. Notably, P-gp inhibition would be expected to increase intracellular drug accumulation and thereby enhance apparent permeability, which is inconsistent with our findings (Lespine et al. 2009; Sharom 2011). In addition, significant modulation of P-gp expression is unlikely under the short experimental conditions used in this study, as transcriptional regulation typically requires longer exposure times (Tolson and Wang 2010). Given the highly lipophilic nature of both EPR and the tested vitamins, it is more plausible that their interaction within the lipid bilayer alters the membrane properties and may promote drug sequestration, thereby reducing its transcellular diffusion (Wang and Quinn 2000; Atkinson et al. 2021; Radzin et al. 2023).

Vitamin A propionate (retinyl propionate) is a lipophilic retinyl ester form of VA, as described in comparative metabolic studies showing its distinct profile among retinoid esters such as retinyl palmitate (Bjerke et al. 2021). Retinyl esters are predominantly transported across Caco-2 monolayers via membrane partitioning and passive transcellular diffusion rather than carrier-mediated uptake, with a tendency for apical retention and limited basolateral efflux (Artursson and Karlsson 1991; Herr et al. 1999; During and Harrison 2007; Volpe et al. 2007). The lack of a comparable effect for VE in our study is consistent with existing literature indicating that P-gp inhibition is primarily associated with synthetic VE derivatives, such as TPGS, rather than natural α-tocopherol (Yu et al. 1999; Yang et al. 2018). While α-tocopherol and its analogues have been demonstrated to modify the membrane dipole potential (MDP) and subsequently influence the ligand-binding capacity of P-gp, our results under short-term exposure conditions support a mechanism driven by physicochemical and membrane-level interactions (Collnot et al. 2006; Davis et al. 2015). Therefore, the observed selectivity for VA may stem from its specific ability to perturb membrane packing or lipid ordering more effectively than natural α-tocopherol within the timeframe of our experiments.

As with other lipophilic compounds with limited oral bioavailability, the intestinal permeability of EPR can be effectively investigated using the Caco-2 model to identify membrane-level and intracellular factors restricting transepithelial transport, independent of classical efflux mechanisms (Wahlang et al. 2011; Ustun Alkan et al. 2014). Similar observations have been reported in studies proposing that fat-soluble vitamins may affect drug absorption and bioavailability through nutrient–drug interaction mechanisms (Renaud et al. 2024). From a veterinary pharmacotherapy perspective, such interactions could compromise therapeutic efficacy, particularly in animals receiving vitamin-enriched diets (Finno 2020).

EPR, a macrocyclic lactone, is known to be influenced by P-gp, and impaired P-gp function may increase systemic and central nervous system exposure, as demonstrated for ivermectin (Geyer et al. 2005; Mealey et al. 2023), particularly in certain dogs with MDR1 mutations. However, EPR is generally considered to have a more favourable safety profile, with limited evidence of species-specific neurotoxicity in the literature (Lespine et al. 2009). In the present study, VA and VE decreased EPR permeability, suggesting that co-administration is unlikely to increase systemic exposure or toxicity risk. Accordingly, the likelihood of clinically relevant neurotoxic effects under these conditions appears low.

Our findings suggest that VA propionate, a commonly used VA ester in feed additives (EFSA 2013), may reduce the intestinal permeability of EPR in Caco-2 cell monolayers. Given the short incubation period (2 h), these effects are more plausibly attributed to acute alterations in membrane fluidity or tight-junction organisation rather than delayed genomic regulation of epithelial transport pathways. These findings emphasise that nutrient-drug interactions should be considered not only from a transporter-based perspective but also in terms of membrane-level physicochemical effects.

In this study, the effects of VA and VE on the intestinal permeability of EPR were evaluated using the Caco-2 cell monolayer model. Our findings demonstrated that both vitamins, particularly VA, reduced the apparent permeability of EPR. These results suggest that the observed reduction in permeability may be attributed to acute, membrane-level physicochemical interactions, such as alterations in membrane organisation or a reduced availability of the freely diffusible drug fraction at the intestinal interface. From a veterinary pharmacological perspective, these results indicate that lipophilic dietary supplements, particularly VA esters, may interfere with the intestinal absorption of orally administered EPR. Such nutrient-drug interactions warrant careful consideration in veterinary practice, as they may influence therapeutic efficacy and residue profiles in food-producing animals. Overall, this study provides experimental evidence supporting the relevance of membrane-mediated nutrient-drug interactions in veterinary pharmacotherapy and highlights the need to consider dietary composition when evaluating the oral use of antiparasitic agents.

## References

[R1] Artursson P, Karlsson J. Correlation between oral drug absorption in humans and apparent drug permeability coefficients in human intestinal epithelial (Caco-2) cells. Biochem Biophys Res Commun. 1991 Mar 29;175(3):880-5. 10.1016/0006-291x(91)91647-u 1673839

[R2] Atkinson J, Marquardt D, DiPasquale M, Harroun T. From fat to bilayers: Understanding where and how vitamin E works. Free Radic Biol Med. 2021 Nov 20;176:73-9. 10.1016/j.freeradbiomed.2021.09.015 34555454

[R3] Awortwe C, Fasinu PS, Rosenkranz B. Application of Caco-2 cell line in herb-drug interaction studies: Current approaches and challenges. J Pharm Pharm Sci. 2014;17(1):1-19. 10.18433/j30k63 PMC4355448 24735758

[R4] Badie C, Lespine A, Devos J, Sutra JF, Chartier C. Kinetics and anthelmintic efficacy of topical eprinomectin when given orally to goats. Vet Parasitol. 2015 Apr 15;209(1-2):56-61. 10.1016/j.vetpar.2015.02.013 25744609

[R5] Berginc K, Zakelj S, Kristl A. In vitro interactions between aged garlic extract and drugs used for the treatment of cardiovascular and diabetic patients. Eur J Nutr. 2010 Sep;49(6):373-84. 10.1007/s00394-010-0095-x 20140680

[R6] Bjerke DL, Li R, Price JM, Dobson RLM, Rodrigues M, Tey C, Vires L, Adams RL, Sherrill JD, Styczynski PB, Goncalves K, Maltman V, Przyborski S, Oblong JE. The vitamin A ester retinyl propionate has a unique metabolic profile and higher retinoid-related bioactivity over retinol and retinyl palmitate in human skin models. Exp Dermatol. 2021 Feb;30(2):226-36. 10.1111/exd.14219 33098193

[R7] Bordes L, Ticoulet D, Sutra JF, Lespine A, Jacquiet P. Lack of efficacy of topical administration of eprinomectin against gastrointestinal nematode in a French dairy sheep farm: A case of underexposure of worms. Vet Rec Case Rep. 2022 Jul;10(4):e435.

[R8] Campbell WC. History of avermectin and ivermectin, with notes on the history of other macrocyclic lactone antiparasitic agents. Curr Pharm Biotechnol. 2012 May;13(6):853-65. 10.2174/138920112800399095 22039784

[R9] Collnot EM, Baldes C, Wempe MF, Hyatt J, Navarro L, Edgar KJ, Schaefer UF, Lehr CM. Influence of vitamin E TPGS poly(ethylene glycol) chain length on apical efflux transporters in Caco-2 cell monolayers. J Control Release. 2006 Mar 10;111(1-2):35-40. 10.1016/j.jconrel.2005.11.005 16410030

[R10] Davis S, Davis BM, Richens JL, Vere KA, Petrov PG, Winlove CP, O’Shea P. alpha-Tocopherols modify the membrane dipole potential leading to modulation of ligand binding by P-glycoprotein. J Lipid Res. 2015 Aug;56(8):1543-50. 10.1194/jlr.M059519 PMC4513995 26026069

[R11] DeHaven WI, Conner DP. The effects of food on drug bioavailability and bioequivalence. In: Yu LX, Li BV, editors. FDA bioequivalence standards. New York (NY): Springer; 2014. p. 95-118.

[R12] During A, Harrison EH. Mechanisms of provitamin A (carotenoid) and vitamin A (retinol) transport into and out of intestinal Caco-2 cells. J Lipid Res. 2007;48(10):2283-94. 10.1194/jlr.M700263-JLR200 17644776

[R13] EFSA. EFSA Panel on Additives and Products or Substances used in Animal Feed (FEEDAP). Scientific opinion on the safety and efficacy of vitamin E as a feed additive for all animal species. EFSA J. 2010 May;8(6):1635. 10.2903/j.efsa.2010.1635 PMC13085184 42006986

[R14] EFSA. EFSA Panel on Additives and Products or Substances used in Animal Feed (FEEDAP). Scientific opinion on the safety and efficacy of vitamin A (retinyl acetate, retinyl palmitate and retinyl propionate) as a feed additive for all animal species and categories. EFSA J. 2013 Jan;11(1):3037. 10.2903/j.efsa.2013.3037 PMC13159827 42124957

[R15] Escriba PV, Gonzalez-Ros JM, Goni FM, Kinnunen PK, Vigh L, Sanchez-Magraner L, Fernandez AM, Busquets X, Horvath I, Barcelo-Coblijn G. Membranes: A meeting point for lipids, proteins and therapies. J Cell Mol Med. 2008 Jun;12(3):829-75. 10.1111/j.1582-4934.2008.00281.x PMC4401130 18266954

[R16] EMEA. European Agency for the Evaluation of Medicinal Products, Veterinary Medicines Evaluation Unit. Eprinomectin: Summary report (1). EMEA/MRL/114/96-FINAL. London: EMEA; 1996.

[R17] Finno CJ. Veterinary pet supplements and nutraceuticals. Nutr Today. 2020 Mar-Apr;55(2):97-101. 10.1097/nt.0000000000000399 PMC7802882 33446942

[R18] Geary TG, Moreno Y. Macrocyclic lactone anthelmintics: Spectrum of activity and mechanism of action. Curr Pharm Biotechnol. 2012 May;13(6):866-72. 10.2174/138920112800399077 22039785

[R19] Geyer J, Doring B, Godoy JR, Leidolf R, Moritz A, Petzinger E. Frequency of the nt230 (del4) MDR1 mutation in Collies and related dog breeds in Germany. J Vet Pharmacol Ther. 2005 Dec;28(6):545-51. 10.1111/j.1365-2885.2005.00692.x 16343287

[R20] Gokbulut C, Di Loria A, Gunay N, Masucci R, Veneziano V. Plasma disposition, concentration in the hair, and anthelmintic efficacy of eprinomectin after topical administration in donkeys. Am J Vet Res. 2011 Dec;72(12):1639-45. 10.2460/ajvr.72.12.1639 22126692

[R21] Gunes Y, Blanco-Paniagua E, Anlas C, Sari AB, Bakirel T, Ustuner O, Merino G. Role of the Abcg2 transporter in plasma, milk, and tissue levels of the anthelmintic monepantel in mice. Chem Biol Interact. 2024 Aug 1;398:111117. 10.1016/j.cbi.2024.111117 38906501

[R22] Hamel D, Visser M, Mayr S, Rauh R, Wang H, Fankhauser R, Rehbein S. Eprinomectin pour-on: Prevention of gastrointestinal and pulmonary nematode infections in sheep. Vet Parasitol. 2018 Dec 15;264:42-6. 10.1016/j.vetpar.2018.11.002 30503090

[R23] Han Y, Tan TM, Lim LY. Effects of capsaicin on P-gp function and expression in Caco-2 cells. Biochem Pharmacol. 2006 Jun 14;71(12):1727-34. 10.1016/j.bcp.2006.03.024 16674925

[R24] Herr FM, Li E, Weinberg RB, Cook VR, Storch J. Differential mechanisms of retinoid transfer from cellular retinol binding proteins types I and II to phospholipid membranes. J Biol Chem. 1999 Apr 2;274(14):9556-63. 10.1074/jbc.274.14.9556 10092641

[R25] Hidalgo IJ, Raub TJ, Borchardt RT. Characterization of the human colon carcinoma cell line (Caco-2) as a model system for intestinal epithelial permeability. Gastroenterology. 1989 Mar;96(3):736-49. 2914637

[R26] Hinney B, Wiedermann S, Kaiser W, Krucken J, Joachim A. Eprinomectin and moxidectin resistance of trichostrongyloids on a goat farm in Austria. Pathogens. 2022 Apr 21;11(5):498. 10.3390/pathogens11050498 PMC9143937 35631019

[R27] Husain A, Makadia V, Valicherla GR, Riyazuddin M, Gayen JR. Approaches to minimize the effects of P-glycoprotein in drug transport: A review. Drug Dev Res. 2022 Jun;83(4):825-41. 10.1002/ddr.21918 35103340

[R28] ICH. International Council for Harmonisation. ICH harmonised tripartite guideline: Validation of analytical procedures: Text and methodology Q2(R1). Geneva: ICH; 1996.

[R29] ICH. International Council for Harmonisation. ICH harmonised guideline: Biopharmaceutics classification system-based biowaivers M9. Geneva: ICH; 2019.

[R30] International Transporter Consortium; Giacomini KM, Huang SM, Tweedie DJ, Benet LZ, Brouwer KL, Chu X, Dahlin A, Evers R, Fischer V, Hillgren KM, Hoffmaster KA, Ishikawa T, Keppler D, Kim RB, Lee CA, Niemi M, Polli JW, Sugiyama Y, Swaan PW, Ware JA, Wright SH, Yee SW, Zamek-Gliszczynski MJ, Zhang L. Membrane transporters in drug development. Nat Rev Drug Discov. 2010 Mar;9(3):215-36. 10.1038/nrd3028 PMC3326076 20190787

[R31] Kiki-Mvouaka S, Menez C, Borin C, Lyazrhi F, Foucaud-Vignault M, Dupuy J, Collet X, Alvinerie M, Lespine A. Role of P-glycoprotein in the disposition of macrocyclic lactones: A comparison between ivermectin, eprinomectin, and moxidectin in mice. Drug Metab Dispos. 2010 Apr;38(4):573-80. 10.1124/dmd.109.030700 20089736

[R32] Koljonen M, Hakala KS, Ahtola-Satila T, Laitinen L, Kostiainen R, Kotiaho T, Kaukonen AM, Hirvonen J. Evaluation of cocktail approach to standardise Caco-2 permeability experiments. Eur J Pharm Biopharm. 2006 Nov;64(3):379-87. 10.1016/j.ejpb.2006.06.006 16914297

[R33] Lennernas H, Palm K, Fagerholm U, Artursson P. Comparison between active and passive drug transport in human intestinal epithelial (Caco-2) cells in vitro and human jejunum in vivo. Int J Pharm. 1996 Jan 2;127(1):103-7.

[R34] Lespine A, Chartier C, Hoste H, Alvinerie M. Endectocides in goats: Pharmacology, efficacy and use conditions in the context of anthelmintics resistance. Small Rumin Res. 2012 Mar;103(1):10-7.

[R35] Lespine A, Dupuy J, Alvinerie M, Comera C, Nagy T, Krajcsi P, Orlowski S. Interaction of macrocyclic lactones with the multidrug transporters: The bases of the pharmacokinetics of lipid-like drugs. Curr Drug Metab. 2009 Mar;10(3):272-88. 10.2174/138920009787846297 19442089

[R36] Losel RM, Falkenstein E, Feuring M, Schultz A, Tillmann HC, Rossol-Haseroth K, Wehling M. Nongenomic steroid action: Controversies, questions, and answers. Physiol Rev. 2003 Jul;83(3):965-1016. 10.1152/physrev.00003.2003 12843413

[R37] McDowell LR. Vitamin nutrition of livestock animals: Overview from vitamin discovery to today. Can J Anim Sci. 2006 Jun;86(2):171-9.

[R38] McKellar QA, Gokbulut C. Pharmacokinetic features of the antiparasitic macrocyclic lactones. Curr Pharm Biotechnol. 2012 May;13(6):888-911. 10.2174/138920112800399194 22039787

[R39] Mealey KL, Owens JG, Freeman E. Canine and feline P-glycoprotein deficiency: What we know and where we need to go. J Vet Pharmacol Ther. 2023 Jan;46(1):1-16. 10.1111/jvp.13102 PMC10092536 36326478

[R40] Miller RA, McCluney TS, Halleran JL, Baynes RE, Foster DM. The pharmacokinetics of subcutaneous eprinomectin in plasma and milk in dry dairy cattle. J Vet Pharmacol Ther. 2025 May;48(3):163-9. 10.1111/jvp.13488 PMC12066900 39460598

[R41] Mohammed AA, Ahmed M. Evidence-based veterinary medicine in developing countries: Challenges and opportunities. Acta Vet Eurasia. 2024 Jan;50(1):83-6.

[R42] Molyneux DH, Bradley M, Hoerauf A, Kyelem D, Taylor MJ. Mass drug treatment for lymphatic filariasis and onchocerciasis. Trends Parasitol. 2003 Nov;19(11):516-22. 10.1016/j.pt.2003.09.004 14580963

[R43] Murri S, Knubben-Schweizer G, Torgerson P, Hertzberg H. Frequency of eprinomectin resistance in gastrointestinal nematodes of goats in canton Berne, Switzerland. Vet Parasitol. 2014 Jun 16;203(1-2):114-9. 10.1016/j.vetpar.2014.02.052 24661808

[R44] Podszun M, Frank J. Vitamin E-drug interactions: Molecular basis and clinical relevance. Nutr Res Rev. 2014 Dec;27(2):215-31. 10.1017/S0954422414000146 25225959

[R45] Radzin S, Wisniewska-Becker A, Markiewicz M, Betkowski S, Furso J, Waresiak J, Grolik J, Sarna T, Pawlak AM. Structural impact of selected retinoids on model photoreceptor membranes. Membranes (Basel). 2023 Jun 1;13(6):575. 10.3390/membranes13060575 PMC10303579 37367779

[R46] Rambozzi L, Torsiello B, Formisano R, Pasquetti M, Molinar Min AR, Giammarino M, Battaglini L, Sangrali M, Renna M. Anthelmintic resistance to pour-on eprinomectin against gastrointestinal strongyles and effects on production parameters in early-lactating dairy goats. Vet Sci. 2025 Nov 15;12(11):1088. 10.3390/vetsci12111088 PMC12656967 41295726

[R47] Rehbein S, Kellermann M, Wehner TA. Pharmacokinetics and anthelmintic efficacy of topical eprinomectin in goats prevented from grooming. Parasitol Res. 2014 Nov;113(11):4039-44. 10.1007/s00436-014-4072-9 25106840

[R48] Reker D, Shi Y, Kirtane AR, Hess K, Zhong GJ, Crane E, Lin CH, Langer R, Traverso G. Machine learning uncovers food- and excipient-drug interactions. Cell Rep. 2020 Mar 17;30(11):3710-6.e4. 10.1016/j.celrep.2020.02.094 PMC7179333 32187543

[R49] Renaud D, Holler A, Michel M. Potential drug-nutrient interactions of 45 vitamins, minerals, trace elements, and associated dietary compounds with acetylsalicylic acid and warfarin-a review of the literature. Nutrients. 2024;16(7):950. 10.3390/nu16070950 PMC11013948 38612984

[R50] Rostang A, Devos J, Chartier C. Review of the eprinomectin effective doses required for dairy goats: Where do we go from here? Vet Parasitol. 2020 Jan;277:108992. 10.1016/j.vetpar.2019.108992 31835054

[R51] Sharom FJ. The P-glycoprotein multidrug transporter. Essays Biochem. 2011 Sep 7;50(1):161-78. 10.1042/bse0500161 21967057

[R52] Stern L, Gwaltney-Brant SM, DeClementi C, Gupta RC. Macrocyclic lactone endectocides. In: Gupta RC, editor. Veterinary toxicology. 4^th^ ed. Amsterdam: Academic Press; 2025. p. 547-62.

[R53] Thomas JA. Drug-nutrient interactions. Nutr Rev. 1995 Oct;53(10):271-82. 10.1111/j.1753-4887.1995.tb01477.x 8584285

[R54] Tolson AH, Wang H. Regulation of drug-metabolizing enzymes by xenobiotic receptors: PXR and CAR. Adv Drug Deliv Rev. 2010 Oct 30;62(13):1238-49. 10.1016/j.addr.2010.08.006 PMC2991607 20727377

[R55] Tras B, Uney K, Parlak TM, Tufan O. Vitamins E and A increase the passing of the P-gp substrate ivermectin into the brain in mice. Can J Physiol Pharmacol. 2023 Sep 1;101(9):475-80. 10.1139/cjpp-2023-0078 37235885

[R56] CDER. US Food and Drug Administration, Center for Drug Evaluation and Research. Guidance for industry: Food-effect bioavailability and fed bioequivalence studies. Silver Spring (MD): FDA; 2002.

[R57] Ustun Alkan F, Anlas C, Cinar S, Yildirim F, Ustuner O, Bakirel T, Gurel A. Effects of curcumin in combination with cyclophosphamide on canine mammary tumour cell lines. Vet Med-Czech. 2014 Nov;59(11):553-60.

[R58] Verhoeckx K, Cotter P, Lopez-Exposito I, Kleiveland C, Lea T, Mackie A, Wichers H, editors. The impact of food bioactives on health: In vitro and ex vivo models. Cham: Springer International Publishing; 2015. 29787039

[R59] Volpe DA, Faustino PJ, Ciavarella AB, Asafu-Adjaye EB, Ellison CD, Yu LX, Hussain AS. Classification of drug permeability with a Caco-2 cell monolayer assay. Clin Res Regul Aff. 2007;24(1):39-47.

[R60] Volpe DA. Drug-permeability and transporter assays in Caco-2 and MDCK cell lines. Future Med Chem. 2011 Dec;3(16):2063-77. 10.4155/fmc.11.149 22098353

[R61] Wahlang B, Pawar YB, Bansal AK. Identification of permeability-related hurdles in oral delivery of curcumin using the Caco-2 cell model. Eur J Pharm Biopharm. 2011 Feb;77(2):275-82. 10.1016/j.ejpb.2010.12.006 21147222

[R62] Wang X, Quinn PJ. The location and function of vitamin E in membranes (review). Mol Membr Biol. 2000 Jul-Sep;17(3):143-56. 10.1080/09687680010000311 11128973

[R63] Yang C, Wu T, Qi Y, Zhang Z. Recent advances in the application of vitamin E TPGS for drug delivery. Theranostics. 2018 Jan 1;8(2):464-85. 10.7150/thno.22711 PMC5743561 29290821

[R64] Yu L, Bridgers A, Polli J, Vickers A, Long S, Roy A, Winnike R, Coffin M. Vitamin E-TPGS increases absorption flux of an HIV protease inhibitor by enhancing its solubility and permeability. Pharm Res. 1999 Dec;16(12):1812-7. 10.1023/a:1018939006780 10644067

